# ePyDGGA: automatic configuration for fitting epidemic curves

**DOI:** 10.1038/s41598-023-43958-2

**Published:** 2024-01-08

**Authors:** Josep Alòs, Carlos Ansótegui, Ivan Dotu, Manuel García-Herranz, Pol Pastells, Eduard Torres

**Affiliations:** 1 Logic and Optimization Group, University of Lleida, Lleida, Spain; 2 Giga, UNICEF, New York, USA; 3 Frontier Data Technologies Unit, UNICEF, New York, USA; 4 Barcelona, Spain

**Keywords:** Computational models, Machine learning, Software

## Abstract

Many epidemiological models and algorithms are used to fit the parameters of a given epidemic curve. On many occasions, fitting algorithms are interleaved with the actual epidemic models, which yields combinations of model-parameters that are hard to compare among themselves. Here, we provide a model-agnostic framework for epidemic parameter fitting that can (fairly) compare different epidemic models without jeopardizing the quality of the fitted parameters. Briefly, we have developed a Python framework that expects a Python function (epidemic model) and epidemic data and performs parameter fitting using automatic configuration. Our framework is capable of fitting parameters for any type of epidemic model, as long as it is provided as a Python function (or even in a different programming language). Moreover, we provide the code for different types of models, as well as the implementation of 4 concrete models with data to fit them. Documentation, code and examples can be found at https://ulog.udl.cat/static/doc/epidemic-gga/html/index.html.

## Introduction

In December 2019 the novel coronavirus SARS-CoV2 (COVID-19) emerged in Wuhan, China. Despite drastic containment measures implemented by the Chinese government, the disease quickly spread worldwide, officially reaching the pandemic level in March 2020^1^. As of December 2022, the number of reported cases rose to 650 million, with nearly 6.6 million deaths^2^.

Mathematical models offer a precious tool for public health authorities to control epidemics . Indeed, models can be used for estimating transmission parameters, understanding contagion dynamics and mechanisms or comparing interventions for containing emerging epidemics^3,4^. Furthermore, epidemic models can be used to obtain short and long-term predictions, which in turn may enable decision-makers to optimize possible control strategies, such as containment measures, lockdowns and vaccination campaigns.

Interest in epidemic forecasting and predictive analytics through modeling has grown dramatically over the last decade, as reflected by the increasing amount of organized challenges to predict epidemics of diseases as diverse as Chikungunya^5^, Dengue fever^6,7^, Influenza^8,9^ or Ebola^10^; the creation of modeling hubs^11^ and operational hubs and platforms of decision makers on disease control like CDC^12^ or WHO^13^ looking to transition from research and innovation to better intelligence and tools to fight epidemics.

When faced with a new epidemic, as contagion data starts being collected, computational epidemiologists are prompted with the task of modeling the spread of the infectious disease and setting the parameters that are intrinsic to such models. Once these models have been generated and their parameters fitted to the available data, they are used to predict the contagion curve, assess the impact of potential interventions, etc. Different models can (and do) lead to different conclusions and their validity is often assessed through how well their parameters explain the data that has been used to fit them. A problematic issue arises in this context when, typically, different types of models are fitted using different approaches and different data even in some cases, when the modeling and the fitting are intertwined.

Broadly speaking, models can be in one dimension, deterministic or stochastic and, in another dimension, compartmental or agent-based. Moreover, models can be classified by the way they treat both time and space, whether discrete or continuous. The combination of all these features results in a vast array of model types that are typically fitted using different approaches: partial differential equations (Laplace), nonlinear least squares fitting, inference (MCMC), Genetic Algorithms, etc. Therefore, it is clear that the comparison of different models with different epidemic parameters becomes a non-straightforward task. At the same time, model results have transcended the expert sphere and are now scrutinized in the public domain^14,15^, potentially affecting public opinion or the decision-makers using them.

Indeed, as Viboud et al. indicate^10^: “Infectious disease forecasting is gaining traction in the public health community; however, limited systematic comparisons of model performance exist”. As mathematical models have become more central to policy and decision-making in the public health community, there has also been a growing debate on their validity and usefulness^16,17^, their general lack of transparency^18^ and the scarce availability of model performance comparisons and tools to do so systematically^10^. This could be further complicated by the growing scientific evidence and interest pointing at model ensembles as best performing strategies for predictive analytics^19,20^.

In this work we propose a new framework (ePyDGGA), based on automatic configuration (AC), that can be seen as model type agnostic, to fit epidemic parameters for any type of epidemic model. Note that model agnostic means that the method to fit the parameters of the model is not dependant on the model or even the type of model, it only needs to know the parameters to be fitted, the data and a (any) cost function. We provide implementations for many different types of models within the framework and we also show how performance (based on the error from the epidemic model prediction against the real data) can be even better than that achieved by more “traditional” fitting methods. In particular, our experiments focus on fitting the epidemic models a posteriori, to better understand the biological variables that define the model. Effectively, we provide a fitting mechanism that can be used in any type of epidemiological model without jeopardizing the quality of the fitted parameters, therefore providing a way to fairly compare (and fit) different models (for the same epidemic curve). Moreover, since the AC process requires high computation power we show how all the computations can be run transparently within a cloud service.

## Preliminaries

For the sake of completion, let us here define some concepts that, although known, will help illustrate our use cases further on.

### Compartmental models: SIR model

The most basic SIR model implementation is straightforward. We have the three compartments (S)usceptible, (I)nfected, and (R)ecovered. In the mean-field approximation, or in the deterministic case, the equations describing the SIR model are:

1 $$\begin{aligned} \begin{aligned} \frac{{d}S(t)}{{d}t}&= -\frac{\beta }{N} I(t) S(t) \\ \frac{{d}I(t)}{{d}t}&= -\delta I(t) + \frac{\beta }{N} I(t) S(t) \\ \frac{{d}R(t)}{{d}t}&= \delta I(t) \end{aligned} \end{aligned}$$

where *S*(*t*), *I*(*t*) and *R*(*t*) are the number of susceptible, infectious and recovered individuals at time *t*; $\beta$ is the infectivity and $\delta$ is the recovery rate. Additionally, $N = S(t) + I(t) + R(t), \forall t$. Note that *N* is different from the real population of the region under analysis, as it is unknown which fraction of the population is susceptible to the pathogen. Thus, usually, *N* is defined as “active population” and set as a model parameter^21^.

The following condition must be fulfilled:

2 $$\begin{aligned} S(t) + I(t) + R(t) = N \end{aligned}$$

A basic Python implementation of the (time continuous variant) SIR model is shown in Listing 1. Essentially, the |sir| function receives the real evolution of the epidemic in the |data| parameter, as well as the active population value *N*, the initial values of *I* and *R* and the values for the $\beta$ and $\delta$ parameters. This function computes the solution (i.e. the evolution of the values in the *S*, *I*, and *R* compartments) of the ODE system shown in Eq. (1) for the given parameters, and returns the Mean Squared Error from the predicted infected cases each day with the real infected cases provided. See Supplementary Listing 1 for a description of the full implementation of the SIR model in Python.

Other models can be easily adapted from this code. In particular, in this work, we consider the variants SIRD, SAIR, SEAIR, SIDARTHE^22^ and SEIPAHRF^23^.
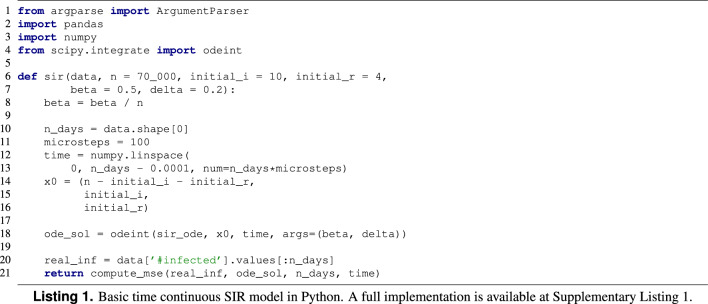


### Automatic configuration algorithms and tools

Consider the scenario where a company or a research group has devised a new algorithm, system, or tool, to which we refer as the *target* algorithm without loss of generality. This algorithm accepts input data, performs a computation or task, and reports a result. Moreover, the algorithm opens to the user a set of parameters that can be set in many different ways that impact the quality of the result. Informally, the algorithm configuration problem is to determine a well-performing parameter configuration of a given algorithm across a given set of instances or input data.

Since we do not know the input data on which the target algorithms will ultimately be run on by the users of these solvers, the developers of these tools have three options: They can either aim to find good default parameters for their solver that work reasonably well, by hand. They can try to write a user manual that explains the solver parameters to the users so that they can search for good parameterizations for their respective applications. Or they can employ an automatic solver configurator that finds decent default parameters, as well as allows users to tune the solver for a particular input data after deployment.

Algorithm tuners were developed to tune and customize solvers so that they would perform better on specific sets of problem instances^24–26^. Moreover, managerial dispatch technologies were invented to choose, at runtime, which solver to run for a given instance^27–31^. And finally, combining self-tuning and selection technologies, methods have been invented to choose a solver parameterization instance-specifically at runtime^32,33^.

#### Definition 1

Let *A* be the target algorithm (that we want to automatically configure) with $\{p_1, \ldots , p_n\}$ parameters of domain $d(p_i)$. These parameters can be *categorical*, which implies they can assume a small, fixed number of values, or *numerical*, which implies they represent rational or integer values. The parameter space of all parameter settings *P* is the subset of $d(p_1) \times \cdots \times d(p_n)$ of *valid* parameter combinations. By $A_{\theta }$ we denote the *instantiation* of algorithm *A* with parameter setting or configuration $\theta \in P$.

#### Definition 2

Let *I* be the set of instances that will be used to configure the target algorithm *A*. In the context of fitting Epidemiologic Models, an instance is the real evolution of the epidemic in a geographical region over time.

#### Definition 3

The *Automatic algorithm configuration problem* is the optimization problem which accepts as input an algorithm *A*, a set of instances *I*, a Performance Metric $PM(A_{\theta }, I)$ which measures the performance of $A_{\theta }$ on the set of instances *I*, and a configuration budget *B* (time limits, memory limits, etc). The objective of the optimization is to find, without exceeding the resources indicated by *B*, a configuration $\theta ^* \in P$ that maximizes the expected performance of *A* over the set of instances *I*.

Fitting an epidemic model (EM) corresponds to minimizing the difference between the real evolution and the predicted evolution by changing the epidemic parameters.

As we want to minimize this difference we define the performance metric as $PM(A_{\theta }, I) = -cost(EM_\theta ,I)$, where *I* is a single instance that contains the real data we want to fit, $EM_\theta$ is the prediction of the Epidemic Model parameterized with the configuration $\theta$, and *cost* is a cost function (e.g. mean square error) that measures the error between the real evolution and the predicted evolution.

## The ePyDGGA framework

**Figure 1:**
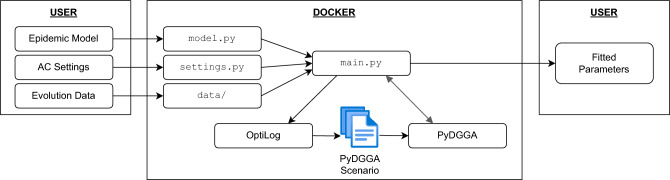
Main components of the ePyDGGA framework.

Here we present ePyDGGA (available at https://ulog.udl.cat/software/). Broadly speaking, it is a framework that allows the user to configure (in this context, to adjust or fit epidemiological parameters) a Python function into a distributed computing platform. Figure 1 depicts the structure and relationship among all the components. Briefly, the user defines their epidemic model in *model.py*, as well as the AC settings in *settings.py*, and specifies the evolution data inside the *data/*folder. Then, *main.py* calls both *settings.py* and *model.py*, loads the provided data and calls OptiLog (available at https://pypi.org/project/optilog/)^34,35^ to produce the necessary files for PyDGGA (available at https://ulog.udl.cat/software/)^36^ to perform automatic configuration (what we call the *PyDGGA scenario*). Finally, it returns the fitted parameters in a readable manner to the user. Below, we explain the main components in more detail.

### Automatic configurator: PyDGGA

Our automatic configurator engine is PyDGGA^36^, a Python tool that implements a distributed version of the automatic algorithm configurator GGA^24^, which is a specialized genetic algorithm to find high-quality parameters for solvers and algorithms. PyDGGA implements GGA using an event-driven architecture and runs a simulation of future generations of the genetic algorithm to maximize the usage of the available computing resources. PyDGGA offers a friendly interface to deploy distributed AC scenarios on shared high-performance computing clusters.

### Embedding epidemic models within OptiLog

The main barrier to using AC tools is that they require a deep knowledge of the tool, due to (1) a large number of settings of the tool itself, and (2) the format of the so-called AC scenario. An AC scenario is the definition of the process that is being tuned, stating how to execute the target algorithm (in our case the epidemic model), the budget for the AC algorithm, and the set of instances to use. Each tool could also require additional information. Setting up the scenario for a tool is a cumbersome and error-prone task, due to the tools not following a standard format. The proprietary scenario definition for each tool also results in a lack of portability among them.

We can remove this barrier using OptiLog^34,35^. OptiLog is a Python framework for rapid prototyping of SAT-based systems. It also offers modules to ease the experimentation workflow, a *Blackbox* module to integrate any software into OptiLog, and a *Tuning* module. For this work, we are interested in these two modules: *Blackbox*, and *Tuning*. In particular, the *Tuning* module will allow us to easily create AC scenarios, and the *Blackbox* module to extend the tuning module to any piece of software, not only Python.

#### Epidemic model implemented in Python

For reference, we take the SIR model explained in “Compartmental models: SIR model” section (Listing 1 and Supplementary Listing 1). The changes that have been done to the original model to be fitted with an AC tool supported by OptiLog are shown in Listing 2. Note that we do not show the internal function implementation as it does not require any change in order to be tuned by OptiLog. The changes, highlighted in red, are the following: first, we declare the |sir| function to be configurable using the decorator |@ac| (line 4), then we add the type and domain of the parameters we want to fit (lines 6–10), and finally we create an entrypoint, which is the function that will load the data provided and report the cost to the AC tool (lines 13–16).
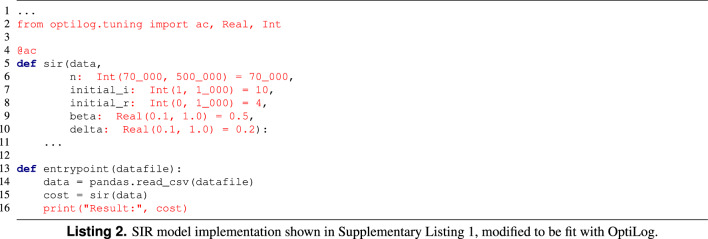


Supplementary Listing 2 shows how the AC scenario is created. OptiLog will automatically generate the scenario with the files in the format required by the specific configurator. The parameters used are defined in Supplementary Table 2.

#### Epidemic model in other languages

If we have an epidemic model implemented in another programming language, we can either use Python bindings for this language or use the *BlackBox* OptiLog’s module. This module allows one to run external scripts and programs in a constrained environment and parse the result.

Assuming we have a (time continuous) SIR model implemented in an executable external program, we present how we could connect the model with OptiLog in Supplementary Listing 3.

We first implement the |BlackBox| (called |ExternalSir|) that represents this executable: First, in line 8 we define the regular expression that will capture the cost of the model from its standard output. Then, we define the parameters in lines 15–21, with the domain annotations. We define where the executable can be found and how it receives the instance (line 26). Finally, we define the format that the executable uses to receive the parameters in lines 32–37.

Once we have the |ExternalSir| created, we create the model function, which will receive an instance of this class. To instruct OptiLog that this instance can be configured automatically, we set the type of the parameter as |CfgObj| (line 40), and then we run the model and report the error.

### Using OptiLog within cloud computing services

While OptiLog allows us to setup the scenario for the AC tool, there is still a barrier on how to install, deploy, and execute it in distributed environments. A Docker image is an executable package for a certain application that contains all the runtime, system tools, libraries, etc., required by the application. It also defines how the application is started, which ports are exposed, which files are available, etc. Usually, those images are created using a *Dockerfile*, which is a definition of the steps to take to create the image. The Docker images are not executed directly but serve as a template to create a container, which is a specific run of the application inside the image.

Our framework allows to not only run the model locally but also to create a Docker^37^ image (although it should work with any open container initiative (OCI) implementation^38^) that can be used to fit the model in the cloud.

### Framework description

**Figure 2:**
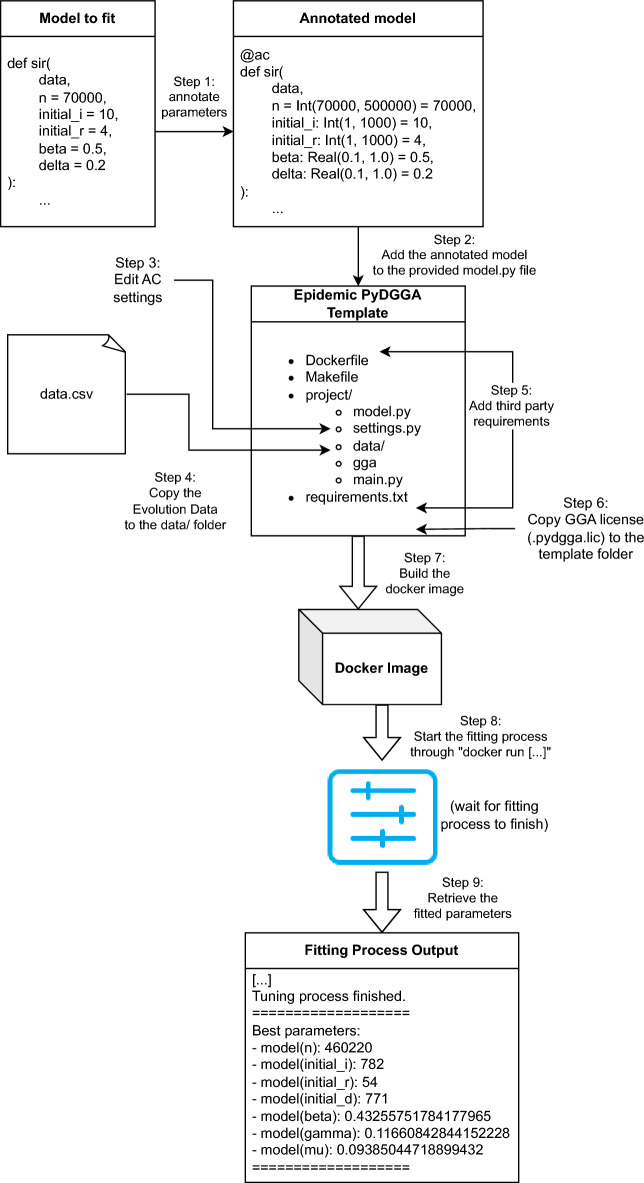
Steps to create and run a new model in ePyDGGA.

Figure 2 shows both our overall framework and the steps to configure an epidemic model taking its Python implementation and relevant data. The template provided is the core of the framework, and the starting point of all the examples provided in https://ulog.udl.cat/static/doc/epidemic-gga/html/index.html and for the results shown in the following section. Let us briefly define each of the parts of the template here:

- A *project* folder. This folder contains, among other files, the *model.py* and *settings.py* files, which will be modified by the user to implement the model and to change the AC tool hyper-parameters (such as the time that the AC tool should run) respectively.
  This folder also contains the *main.py* file, although the user should not modify this file. This file will be used as the entrypoint of the project, to run the model or start the fitting process.
- A *data* folder, inside the *project* folder. It is intended to contain the evolution data to fit the epidemic model.
- A *requirements.txt* file. The user should specify the Python packages that are required to run the model. This is mandatory if the user wants to use Docker.
- A *Dockerfile*. This file will instruct Docker how to create an image with the model and the data ready to be fit. The user has to modify this file only if the model requires software that cannot be installed through a Python package.
- A *Makefile*. This file allows to use the “make” command to build and publish the Docker image for the template.

To start calibrating an epidemic model using the provided template, the first step is to add the epidemic model implementation inside the *model.py* file. In this file, there are two functions: model and entrypoint. To fit the SIR model explained in “Compartmental models: SIR model” and “Embedding epidemic models within OptiLog” sections, we use the “entrypoint” function as in Listing 2 (adding the additional “seed” parameter that for this model we ignore) and the “sir” function as our “model” function. Note that we do not need to set the code to create the AC scenario, as it is managed automatically based on the *settings.py* file (which the user can also modify). Additionally, the user must provide the data to fit the model inside the *projects/data* folder (*Step 4* in Fig. 2).

Then, we would modify the *requirements.txt* and *Dockerfile* to add the required software dependencies of our model.

Finally, we would copy the PyDGGA license file to the template directory with the name “*.pydgga.lic*”. This is required to build the Docker image.

Once the template is ready, to build the Docker image (*Step 7)* we use the following command:
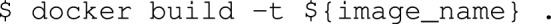


Note that, at this point, the Docker image is ready to be uploaded to some cloud computing service in order to be executed.

To start the fitting process, run:
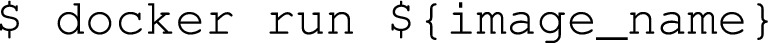


Everything listed after the image name will be considered as parameters that are received by *project/main.py*, the script that runs the template. For example, to list all the possible parameters of the model run:
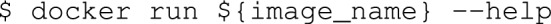


If no parameter is specified to Docker, by default it will start the fitting process expecting one dataset in *project/data* and spawning 4 concurrent processes.

## Results

### Epidemic data sources

We used the data provided by Johns Hopkins University in their Covid-19 data repository^39^, which contains data for over 250 countries. As some countries are reported in a more granular level (province, state...) in three different tables (cumulative confirmed, deaths, and recovered), we aggregate all the data in a time series for each country and extract the infected per day using the cumulative minus the recovered and dead data.

We also use the data in the original papers when comparing with those, as explained in each experiment in “Assessing the impact of AC tools for epidemic models” section. In particular, when comparing with^21,40^ we use the official data from Italian *Protezione Civile*^41^, and reports from WHO^1^ and Worldometer^42^ when comparing with^23^.

### Choosing the AC tool

The first experiment we performed is to compare different non-commercial AC tools in order to choose the most suitable one for tuning epidemic models. In particular, we compare PyDGGA^36^ and SMAC^43^ by fitting the epidemic evolution of 7 epidemic models and 6 countries, for a total of 42 combinations.

In particular, we fit the models SIR, SIR-Erlang, SIR-Network, SIRD, SAIR, SAIR-Erlang, and SEAIR; with the countries China, France, Greece, Iran, Italy, and Turkey. Each model was fit for 54 days (the starting day differs as the countries started to be impacted at different moments).

For each combination of model-country, we set an AC scenario with the time series of the country being the only instance to be used during the tuning process, and setting the AC tools to tune it using 16 parallel processes and a time budget of 1 day for the whole tuning process and 1200 s for each execution of a configuration.

SMAC was configured to use the default parameters, while PyDGGA required to change the mutation probability due to the small number of parameters for the models, setting it to $\frac{1}{n}$, where *n* is the number of parameters of the model, in order to force one parameter to mutate.

Supplementary Table 3 shows the best cost found by PyDGGA and SMAC for each pair of country-model. As we see, PyDGGA is able to fit better the different models and therefore it is the AC tool we selected for the framework.

### Assessing the impact of AC tools for epidemic models

The second type of experiments that we conducted is to evaluate if using the selected AC tool, PyDGGA, we can refine the reported parameters for a model. To do so, we fit the same model as in the original works and compare the reported parameters with the ones found by PyDGGA.

We choose four compartmental models from the literature, in order to obtain sensible parameters from an epidemiological point of view.

First, we study two different SIRD models, as used by Fanelli and Piazza in^44^ and Calafiore et al.^21^. Secondly, we use the SIDARTHE model, introduced by Giordano et al. in^40^. Finally, we test against the SEIPAHRF model presented by^23^. All four models are of different nature, with a different amount of parameters, and all fitted using different approaches, proving the generality of our method. The first one is a stochastic model, the second one is a nonlinear matrix equation, and the third and fourth are composed of ODEs. We compare all of them with the datasets used in their respective papers.

Supplementary Table 2 shows the GGA parameters used in each use case.

#### SIRD model (1)

The authors in^44^ use a stochastic differential evolution algorithm to find the best-fit parameters for the model using data corresponding to the period between January 22nd 2020 and March 15th 2020 (included) They provide results for Italy and China. In the case of Italy, the first day to be fitted is February 11th.

This stochastic model computes a sequence of random evolutions of the epidemic and then averages them to find the predicted evolution. As this process is slower than non-stochastic models, we have long-running executions that might lead to PyDGGA exploring only a small region of the parameters domain. To resolve this, during the configuration process, we reduce the number of evolutions that the algorithm simulates to 10. Then, when the best parameters are found, we execute the model with 1 000 simulations, which is the result presented here.

The solution’s cost is then computed as the sum of squared differences between the solution (averaging over realizations) and the data points for infected, recovered and dead individuals, i.e.

3 $$\begin{aligned} cost = \left( Cost_I + Cost_R + Cost_D\right) / 10^6 \end{aligned}$$

In Table 1 we show the results of this experiment for Italy and China. The “Reported” columns show the values of the parameters reported in^44^, and the columns “GGA” show the values for the parameters fit using PyDGGA. We also show the cost (sum of squared differences) for those values of the parameters. For Italy, all the initial conditions but $S_0$ are fixed.

**Table 1:** Best fit parameters for the stochastic SIRD model in^44^, with an infection rate $r = \beta /N$, a recovery rate $a=\delta (1-\theta )$, and a death rate $d=\delta \theta$.

Parameter	Italy		China	
	Reported	GGA	Reported	GGA
$r (days^{-1})$	$7.9\times 10^{-6}$	$4.0\times 10^{-7}$	$3.95\times 10^{-6}$	$3.89\times 10^{-6}$
$a (days^{-1})$	$2.13\times 10^{-2}$	$3.87\times 10^{-2}$	$3.57\times 10^{-2}$	$3.83\times 10^{-2}$
$d (days^{-1})$	$1.63\times 10^{-2}$	$3.09\times 10^{-2}$	$3.10\times 10^{-3}$	$2.95\times 10^{-3}$
$S_0 (10^4)$	4.13	85.6	8.33	8.52
$I_0$	*Fixed*	*Fixed*	430	422
$R_0$	*Fixed*	*Fixed*	10	4
$D_0$	*Fixed*	*Fixed*	15	5
Cost *I*	245	189	3009	2950
Cost *R*	3	6	3081	3144
Cost *D*	1	3	10	13
Cost	250	199	6699	6031

The values reported in the original paper are shown in columns “Reported” and the values found using PyDGGA are shown in columns “GGA”. The cost values are the sum of squared differences, divided by $10^6$ for visualization purposes.

Note that for China the costs are significantly large (over 6000) with respect to Italy (around 200). This is a direct result of fitting the model over more days (55 days for China and 35 for Italy).

In general, we observe how in both scenarios the GGA is able to improve the overall costs of the reported models (20% for Italy and 10% for China).

#### SIRD model (2)

Calafiore et al.^21^ use a discrete approach for the SIRD model. They also use an additional parameter, *q*, which is a proportionality factor relating the detected number of positives with the actual number of infected.

Instead of fitting the whole function with initial conditions, they use the daily Italy data^41^ for the *I*, *R*, and *D* compartments to obtain *S* in the following way:

4 $$\begin{aligned} S(t) = q \times Pop - I(t) - R(t) - D(t), \end{aligned}$$

where *t* is the time in days, *q* is the proportionality factor, and *Pop* is the real population. Note that fitting *q* is equivalent to fitting *n*, as $n = q \times Pop$.

Then, they compare the real daily change for each compartment (*y*), with the one predicted by the model ($y'$). Thus, there are a total of 4 configurable parameters in this model: 3 epidemic rates: $\beta$, $\delta$ and $\theta$; and one for initial conditions *q*.

They use the following cost function:

5 $$\begin{aligned} cost = \frac{1}{T}\sum _{t=0}^{T-1}w^{T-t}||y(t)-y(t)'||^2 = \sum _C cost_C \end{aligned}$$

where *w* is the so-called forgetting factor, $w \in (0,1]$, and *T* is the number of days. $cost_C$ is the cost associated to a single compartment $C=\{S,I,R,D\}$.

In Table 2 we show the costs and configurations reported, as well as the ones obtained by PyDGGA, for three different ranges: an early stage (from February 24th to March 27th 2020), a late stage (from March 27th to May 18th 2020), and the whole period.

**Table 2:** Best fit parameters and cost for the SIRD model for the three stages analysed in^21^, with an infection rate $r = \beta /N$ and a recovery rate $a=\delta (1-\theta )$.

Parameters	Early stage		Late stage		Whole period	
	Reported	GGA	Reported	GGA	Reported	GGA
*q*	0.011	0.002	0.014	0.005	0.0137	0.0050
$\beta$	0.123	0.200	0.012	0.057	0.0142	0.0637
*a*	0.018	0.013	0.038	0.020	0.0302	0.0194
*d*	0.014	0.011	0.002	0.004	0.0025	0.0054
*w*	0.9	0.9	0.7	0.7	0.9	0.9
Cost *S* ($10^3$)	457	113	531	16	1233	182
Cost *I* ($10^3$)	314	88	1582	13	1989	149
Cost *R* ($10^3$)	26	26	410	16	253	32
Cost *D* ($10^3$)	4	6	9	1	21	5
Cost ($10^3$)	800	234	2535	46	3496	368

The values reported in the original paper are shown in columns “Reported” and the values found using PyDGGA are shown in columns “GGA”. The cost is computed using Eq. (5), and presented divided by $10^3$ for visualization purposes.

In our code, we set $Pop = 59.3\times 10^{6}$, approximately the population of Italy. In the three cases studied, we obtain a substantial improvement with respect to the cost function. The results can be easily interpreted in this case, given that for the three stages, we obtain a higher infectivity ($\beta$) and lower effective population (*q*), as the virus was spreading faster, albeit on a smaller social group.

#### SIDARTHE model

SIDARTHE^40^ is a mean-field deterministic compartmental model. It consists of a series of 8 ODEs. Giordano benchmarks the model with real data from Italy, the same one from the previous section^41^, for the period between February 20th and April 5th 2020, allowing some parameters to change within given intervals, to account for mobility restrictions and social distancing.

We use the same fitting strategy, setting different rates at 6 different times, and allowing only the same 29 parameters used in^40^ to change.

The cost is computed as the sum of squared differences for the infected individuals with different severities (compartments D, R and T), and the recovered individuals H.

6 $$\begin{aligned} cost = \left( Cost_{D} + Cost_{R} + Cost_{T} + Cost_{H} \right) / 10^{6} \end{aligned}$$

As shown in Table 3 we find a better overall solution, improving on three of the four compartments, with the biggest difference in the Diagnosed (D) compartment. For the sake of space, we do not show the values for the 29 configurable parameters. At day 1 the parameters are set as $\alpha =0.594$, $\beta =\delta =0.016$, $\gamma =0.524$, $\epsilon =0.100$, $\theta =0.313$, $\zeta =\eta =0.126$, $\mu =0.010$, $\nu =0.029$, $\tau =0.016$, $\lambda =\rho =0.038$ and $\kappa =\xi =\sigma =0.013$. After day 4, $\alpha =0.332$, $\beta =\delta =0.006$ and $\gamma =0.359$. After day 12, $\epsilon =0.139$. After day 22, $\alpha =0.352$, $\beta =\delta =0.005$ and $\gamma =0.313$; also, $\zeta =\eta =0.038$, $\mu =0.005$, $\nu =0.011$, $\lambda =0.085$ and $\rho =\kappa =\xi =\sigma =0.014$. After day 28, $\alpha =0.225$ and $\gamma =0.089$. Finally, after day 38, $\epsilon =0.241$, $\rho =\kappa =\xi =0.023$, $\sigma =0.011$ and $\zeta =\eta =0.013$.

**Table 3:** Cost of the model SIDARTHE for the different infected compartments (D, R, T, H) for the parameter values reported in^22^ (row “Reported”) and the values found using PyDGGA (row “GGA”).

	$Cost_D$	$Cost_R$	$Cost_T$	$Cost_H$	*cost*
Reported	432	82	33	74	622
GGA	34	82	32	7	156

The costs are computed using Eq. (6).

#### SEIPAHRF model

SEIPAHRF^23^ is also a mean-field deterministic compartmental model. It consists of a series of 8 ODEs. The data is benchmarked against the Wuhan reported by WHO^1^ and Worldometer^42^, between January 4th and March 9th 2020. The total of individuals used in the original paper is 44,000 instead of the 11 million total population, due to the quarantine applied that restricted the movement and limited the spread of the disease.

The results are shown in Table 4. In order, we show the fitted parameters and the mean squared error of the confirmed (I + P + H compartments) individuals for the original paper, for GGA fitted without an initial configuration (GGA (NIC)), for GGA with the reported parameters as an initial configuration (GGA (IC)), and for GGA without an initial configuration and letting the initial exposed, infected, and superspreaders amount to be fitted as well (GGA (NF/NIC)).

**Table 4:** Fitted parameters for the SEIPAHRF model.

Parameter	Reported	GGA (NIC)	GGA (IC)	GGA (NF/NIC)
$\beta$	2.55	4.54	2.93	3.76
*l*	1.56	1.03	2.62	0.9
$\beta '$	7.65	9.1	9.55	1.95
$\kappa$	0.25	0.32	0.14	0.62
$\rho _1$	0.580	0.49	0.31	0.027
$\rho _2$	0.001	0.05	0.14	0.34
$\gamma _a$	0.94	0.61	0.85	0.73
$\gamma _i$	0.27	1.42	0.48	0.8
$\gamma _r$	0.5	0.14	0.27	0.14
$\delta _i$	3.5	4.92	1.95	3.87
$\delta _p$	1	0.95	1.26	0.91
$\delta _h$	0.3	0.65	0.05	0.14
$I_e$	(0)	(0)	(0)	20
$I_i$	(1)	(1)	(1)	15
$I_p$	(5)	(5)	(5)	7
Cost	15.098	4.41	4.17	3.74

Parameters in parenthesis are fixed from the start. The values reported in the original paper are shown in column “Reported” and the values found using PyDGGA are shown in columns “GGA”. The cost is computed using Eq. (5), and presented divided by $10^3$ for visualization purposes.

## Discussion

Epidemic modeling is a complex and sensitive task with multiple and diverse challenges^45^: from model selection itself, that is finding a model complex enough to reflect what is happening but simple enough not to get lost in unnecessary and noisy details; to parameter estimation or model fitting. Talking about “the best model” is normally a blurred concept encompassing performance in many of these different dimensions of goodness.

There are also very different purposes behind model design and/or use: from evaluating the impact (or potential impact) of events and interventions to scenario analysis or forecasting. Different models require different types, quantity and granularity of data. Data quality and availability vary among countries and communities and data bias and diversity can impact model performance^46^. It is difficult to make performance assumptions of a model based on cross-country claims or to weigh the evidence of models using different datasets^47^.

Comparing models is a non-trivial, complex problem^48^, currently entangled to parameter fitting. Modeling approaches are diverse, each with its strengths and weaknesses: compartmental models, network models, agent base models, deterministic, stochastic, micro-simulation based...And parameter values can drastically affect model performance in the same way that model appropriateness can limit performance, regardless of parameter accuracy. In^49^ words: “The ability of a model to make accurate predictions depends not only on the fidelity with which the model represents real-world transmission dynamics but also on the appropriate specification of model parameters and the accuracy of model state variable estimation at the start of a forecast”.

In the absence of a systematic approach, model selection is still very limited^50,51^ and/or manual^7,52^; linked often to the trust on and reputation of the particular research group responsible for the model, and many times to just comparing the final results (or fit) of a model with a specific set of parameters.

In this work, we have explored how automatic configuration tools can help systematize, optimize and solve some of these fundamental problems. First, in fitting model parameters, we have shown how automatic configuration can minimize the burden of modelers, allowing them to focus on modeling itself and in establishing credible ranges and constraints that must be followed by the parameters. In our results, ePyDGGA was able to find combinations of parameters achieving a better fit of the model than with the parameter values supplied by the modelers.

This systematic approach also enables an objective and at-scale tool for comparing models, independent of how good were the modeling teams on finding the best parameter values for their models. automatic configuration tools, when provided with a trusted model, can help in exploring the potential nature of a disease through the observed epidemic, that is, providing a thorough and optimal exploration of what combinations of properties of the epidemic, expressed as parameters, better explain what is being observed. This is of particular importance in modeling emerging diseases^53^, where being able to scrutinize several models and understand the properties of the diseases is especially difficult. As stated by^53^ “Mechanistic models play an essential role in the response to emergence events. However, given the unknowns inherent in the situation, accurately characterizing an emerging pathogen is hard and always will be”.

## Conclusions

We have presented a new framework ePyDGGA that allows users to create new epidemic models and fit them in such a way that model and fitting are clearly separated and thus, comparison among models can be done fairly and without jeopardizing the quality of the fitted parameters.

We have shown that ePyDGGA is suitable for fitting any type of model, even stochastic ones (where we cannot use numerical methods to solve them). We also provide many examples of each of these models within the framework.

Finally, we have shown that by using Docker we can leverage cloud technologies to perform the heavy computation task of exploring the search space of the parameters for an epidemic model.

In terms of future work, we are aware that there might be a need for more complexity in the parameters, for example: (1) relationships among the parameters, i.e., it can be that parameter *A* cannot take value *a* if parameter *B* takes value *b*; (2) parameters might need to be specified in terms of probabilistic functions and not just ranges, i.e., parameter *A* might need to be defined from a Gaussian distribution with mean $\mu$ and standard deviation $\sigma$. Therefore, it is clear that work needs to be done to address these complexities within the framework so they are taken into account within the AC process.

Finally, as it has been argued repeatedly, deciding that a model is *“better”* than others usually depends on several factors or, in optimization terms: it depends on several cost functions. It is naturally better than a model that fits more accurately the observed data. But if an epidemic has been going on for over a year and has moved over several waves or peaks already, it might be preferable that the model fits better the last part of the epidemic, the current one, even if that means not doing so well in fitting the now long gone first wave. A balance might be the preferred choice of an informed decision-maker: *“Whichever model fits best the last part of the epidemic and it reasonably fits the curve corresponding to the whole year”*. Sometimes a decision maker might be more interested in looking at absolute numbers: *“How many people can I expect to become infected...”*. Sometimes only relative size is important: *“Is the peak over? Or should I expect a rebound?”*. Computer Science –through optimization and automatic configuration– offers powerful tools to work with multi-objective optimization problems, allowing different cost functions to be optimized at the same time, and therefore yielding a Pareto Front of solutions (and therefore of fitted parameters) instead of just one. Decision makers could then explore Pareto Fronts, understanding where the boundaries of accuracy are as well as how those boundaries are characterized (e.g. how fast accuracy on a given factor drops when trying to improve accuracy on a different one). We believe this direction to be one of the most natural and powerful ways to continue the work described in this paper in the near future.

### Supplementary Information

Supplementary Information.

## Data Availability

The data used in this study is available in the original repositories: · Covid-19 data repository from Johns Hopkins University^39^ · Italian *Protezione Civile* official data^41^ · WHO reports^1^ · Worldometer^42^.
