# Supplementary material for: ePyDGGA: automatic configuration for fitting epidemic curves
Source: Sci Rep. 2024 Jan 8;14:784. doi: 10.1038/s41598-023-43958-2 (PMC10774272; doi:10.1038/s41598-023-43958-2)
Supplement: Supplementary file 1 — Supplementary Information. [file 41598_2023_43958_MOESM1_ESM.pdf]

ePyDGGA: Automatic Configuration for fitting epidemic curves - Supplementary Material

Josep Alòs[1,*,+], Carlos Ansótegui[1,+], Ivan Dotu[2,+], Manuel García-Herranz[3,+], Pol Pastells[+], and Eduard Torres[1,+]

[1]Logic and Optimization Group, University of Lleida (Spain)
[2]Giga, UNICEF (USA)
[3]Frontier Data Technologies Unit, UNICEF (USA)
[*]Corresponding author. josep.alos@udl.cat
[+]These authors contributed equally to this work

ABSTRACT

Supplementary material for “ePyDGGA: Automatic Configuration for fitting epidemic curves”

1 SIR Model in Python

In this section, we show how to prepare an already implemented Python model to be configured using OptiLog and PyDGGA.

```
from argparse import ArgumentParser
import pandas
import numpy
from scipy.integrate import odeint

def sir(data, n = 70_000, initial_i = 10, initial_r = 4,
        beta = 0.5, delta = 0.2):
    beta = beta / n

    n_days = data.shape[0]
    microsteps = 100
    time = numpy.linspace(
        0, n_days - 0.0001, num=n_days*microsteps)
    x0 = (n - initial_i - initial_r,
          initial_i,
          initial_r)

    ode_sol = odeint(sir_ode, x0, time, args=(beta, delta))

    real_inf = data['#infected'].values[:n_days]
    return compute_mse(real_inf, ode_sol, n_days, time)

def sir_ode(x, t, beta, delta):
    S, I, R = x
    dS_dt = -beta * S * I
    dI_dt = beta * S * I - delta * I
    dR_dt = delta * I
    return dS_dt, dI_dt, dR_dt

def compute_mse(real_infected, ode_sol,
                n_days, timestamps):
    pred = numpy.zeros(n_days)
    for d in range(n_days):
        first_for_day = numpy.searchsorted(timestamps, d)
        pred[d] = ode_sol[first_for_day, 1]

    return ((real_infected - pred)**2).sum() / n_days

def main():
    parser = ArgumentParser()
    parser.add_argument("data")
```

```python
42     parser.add_argument("--n", type=int, required=True)
43     parser.add_argument("--initial-i", type=int, required=True)
44     parser.add_argument("--initial-r", type=int, required=True)
45     parser.add_argument("--beta", type=float, required=True)
46     parser.add_argument("--delta", type=float, required=True)
47     args = parser.parse_args()
48
49     data = pandas.read_csv(args.data)
50     cost = sir(data, args.n, args.initial_i, args.initial_r,
51                args.beta, args.delta)
52     print("MSE:", cost)
53
54 if __name__ == "__main__":
55     main()
```

Supplementary Listing. 1. Full implementation of the basic time continuous SIR model in Python

Supplementary Listing 1 shows the full implementation in Python of the basic SIR model described in Main Section 2.1 and Main Listing 1.

We assume that we have a dataset with the real evolution of the epidemic with as many rows as days we want to simulate and a column “#infected” with the number of people in the Infected compartment at each day (other columns are ignored). For the sake of simplicity, we also assume that we want to fit all the days on the dataset, although this can also be parameterized.

We implement the model in the function `sir`. First, in line 8 we normalize the β parameter to represent $\frac{\beta}{N}$, as seen in Main Eq. 1. Then, we set that, for each day, we want to simulate the interaction between the compartments 100 times, and create an array that represents all the t values to simulate for (lines 10-13). We also define in line 14 the initial values for each compartment.

We define the Ordinary Differential Equation (ODE) system in the function `sir_ode` (line 23). This function receives an array x, which has the values for each compartment at the start of this time step, the current time (not used as the current model is only dependant on the state of the compartments and the β and δ), and the β and δ parameters; and computes the variation of each compartment and returns those variations.

Using *SciPy*[1] `odeint` method (line 18), we integrate the ODE system and receive a matrix of $n\_days \times n\_compartments$, which represents the evolution of each compartment during the simulation.

Finally, in line 21, we compute the mean squared error (MSE) between the simulated infected and the real infected using the `compute_mse` function (line 30) and return it.

```
1  import ...
2  from pathlib import Path
3  from optilog.tuning.configurators import GGAConfigurator
4
5  @ac
6  def sir(...):
7      ...
8
9  def entrypoint(datafile):
10     data = pandas.read_csv(datafile)
11     cost = sir(data)
12     print("Result:", cost)
13
14 def create_scenario(scenario_path):
15     data_folder = Path(__file__).parent / ".." / "data"
16     data_to_fit = [str((data_folder / "demo.csv").resolve())]
17     configurator = GGAConfigurator(
18         entrypoint,
19         global_cfgcalls=[sir],
20         input_data=data_to_fit,
21         run_obj="quality",
22         data_kwarg="datafile",
23         quality_regex="^Result: ([+-]?\d+(?:\.\d+)?)",
24         cost_min=0,
25         cost_max=2<<63,
26         cost_tolerance=0,
27         seed=42,
28         eval_time_limit=3600, # in seconds
29         memory_limit=3 * 1024, # in MB
30
31         # additional settings for this particular configurator
32         min_generations=300,
33         generations=500,
34         mutation_probability=0.3,
35     )
36
37     configurator.generate_scenario(scenario_path)
```

Supplementary Listing. 2. Creation of the GGA scenario for the SIR model in Main Listing 2

Supplementary Listing 2 shows the modifications to automatically configure the model in Supplementary Listing 1.

First, we must annotate the parameters of the `sir` function and provide the `entrypoint` function as shown in Main Listing 2. Next, we define the `create_scenario` function where we provide to the `GGAConfigurator` class all the required information to generate the PyDGGA scenario, such as the function that we want to automatically configure (lines 18 and 19) and the input data (line 20), as well as time and memory limits (lines 28 and 29) and other parameters specific to the PyDGGA (lines 32- 34). The description of the parameters is explained in Supplementary Table 1. Notice that as we explained in Main Section 3.4 the final user does not need to implement this part of the code, as this is automatically handled by the ePyDGGA template.

```
1 ...
2 from optilog.blackbox import *
3 from optilog.running import ParsingInfo
4 from optilog.tuning import *
5
6 class ExternalSir(SystemBlackBox):
7     @staticmethod
8     def get_output_parser():
9         parser = ParsingInfo()
10        parser.add_filter(
11            "cost", r"MSE: ([+-]?(?:\d+(?:\.\d*)?|\.\d+)(?:[eE][+-]?\d+)?)",
12            cast_to=float)
13        return parser
14
15    config = {
16        "n": Int(70_000, 500_000, default=70_000),
17        "initial-i": Int(1, 1_000, default=40),
18        "initial-r": Int(0, 1_000, default=4),
19        "beta": Real(0.1, 1.0, default=0.7),
20        "delta": Real(0.01, 1.0, default=0.1),
21    }
22
23    def __init__(self, *args, **kwargs):
24        _model = (Path(__file__).parent / "sir").resolve()
25        super().__init__(
26            arguments=[_model, SystemBlackBox.Instance],
27            *args,
28            parsing_info=self.get_output_parser(),
29            **kwargs,
30        )
31
32    def format_config(self, args):
33        other = ""
34        for k, v in self.configured.items():
35            other += f" --{k} {v}"
36        args = args + shlex.split(other)
37        return args
38
39 @ac
40 def model(datafile, sir: CfgObj(ExternalSir)):
41     sir.run(datafile)
42     return sir.cost
```

Supplementary Listing. 3. External SIR model

Finally, Supplementary Listing 3 shows how to use OptiLog's *BlackBox* module to configure an external SIR model that might not be implemented in Python. We first implement the `BlackBox` (called `ExternalSir`) that represents this executable: First, in line 8 we define the regular expression that will capture the cost of the model from its standard output. Then, we define the parameters in lines 15-21, with the domain annotations. We define where the executable can be found and how it receives the instance (line 26). Finally, we define the format that the executable uses to receive the parameters in lines 32-37.

Once we have the `ExternalSir` created, we create the model function, which will receive an instance of this class. To instruct OptiLog that this instance can be configured automatically, we set the type of the parameter as `CfgObj` (line 40), and then we run the model and report the error.

2 PyDGGA options

Option	Description
`entrypoint`	Specifies the target function that will be called to start the model. In our case, it is the `entrypoint` function in Supplementary Listing 2.
`global_cfgcalls`	Specifies which functions called by the target (including itself) have configurable parameters. In the example, the function `entrypoint` does not have configurable parameters, but it calls the function `sir` that has configurable parameters, so we declare `sir` as one of the configurable functions.
`input_data`	The instances to be configured with. In our case, we only have one.
`run_obj`	PyDGGA allows setting the PM relative to the runtime or a quality reported by the algorithm. In our case, we want to maximize the quality of the model (i.e minimize the fitting error).
`data_kwarg`	The name of the parameter that receives the instance in the `sir` function.
`quality_regex`	The regular expression that captures the quality metric reported by our function. We match for a string starting with "Result: " followed by a decimal value.
`cost_min`	The minimal cost of our algorithm. The best error is no error (0).
`cost_max`	The maximal cost of our algorithm. As we don't know it in advance, we set it to the largest possible value in Python.
`cost_tolerance`	When PyDGGA finds that the cost does not improve below this tolerance, stops the algorithm. As it is a percentage relative to the maximum and minimum costs, but we don't know the maximum, we set this tolerance to 0.
`seed`	The seed used by PyDGGA internal random decisions.
`eval_time`	The maximum time that PyDGGA is allowed to tune the target. After this time, it will report the best configuration found.
`memory_limit`	The maximum memory that each execution of the target algorithm is allowed to use.
`min_generations`	The minimum number of generations that PyDGGA will run. As each execution of the time continuous SIR model is fast, we can force PyDGGA to run a large number of generations in the given time to refine further the results.
`generations`	The maximum number of generations that PyDGGA will run.
`mutation_probability`	The probability of the mutations during the creation of the new generation. To ensure that at least one parameter is mutated, this probability has to be no less than $\frac{1}{n}$, where n is the number of parameters for the target algorithm. In the example, we have five parameters so it is set to 0.3.

Supplementary Table. 1. Description of PyDGGA options

3 Additional experiments information

Parameter	SIRD 1	SIRD 2	SIDARTHE	SEIPAHRF
Number of generations	60	500	500	500
Minimum generations	50	50	490	490
Population	50	50	50	50
Tuning time limit	1 day	1 day	1 day	1 day

Supplementary Table. 2. GGA parameters used in each use case

country	AC tool	SAIR	SAIR-E	SEAIR	SIR	SIR-E	SIR-N	SIRD
China	PyDGGA	768.29	534.21	8154.10	748.81	1858.37	802.20	6552.68
	SMAC	1105.83	1196.05	9856.06	2889.05	5764.93	2178.35	6599.49
France	PyDGGA	352.55	267.59	1510.61	22.59	436.44	1488.40	127034.04
	SMAC	248.78	174.83	2286.09	161.87	301.78	2773.93	128015.54
Greece	PyDGGA	3.82	4.35	22.27	0.41	0.25	0.50	133622.42
	SMAC	3.96	3.46	26.56	1.64	1.28	8.19	134466.70
Iran	PyDGGA	283.32	159.48	1524.68	199.31	206.00	444.82	44798.58
	SMAC	293.45	325.31	3073.00	457.41	637.52	1350.93	45710.03
Italy	PyDGGA	330.03	342.55	19645.94	73.32	144.58	17755.36	113343.06
	SMAC	371.16	3439.84	42321.17	449.65	582.60	36964.62	114330.95
Turkey	PyDGGA	141.45	306.74	10823.32	120.30	1385.22	13282.43	70374.83
	SMAC	379.65	547.23	13662.59	459.33	33454.07	20771.87	71466.46
# Wins	PyDGGA	5	4	6	6	6	6	6
	SMAC	1	2	0	0	0	0	0
Mean cost improvement using PyDGGA		-70,56	-678,63	-4924,09	-542,37	-6118,55	-5045,70	-810,59

Supplementary Table. 3. Fitted models in PyGGA and SMAC for different countries. Models with the suffix "-E" are erlang distributed models, and models with the suffix "-N" are a network-based variant of the model. The costs are MSE. Each model was fit for 54 days (the starting day differs as the countries started to be impacted at different moments).
SMAC was used with the default settings. For PyDGGA we changed the mutation probability due to the low amount of parameters to fit (we set this value to be at least $\frac{1}{n}$, where n is the number of parameters of the model, in order to force one parameter to mutate).

References

1. Virtanen, P. *et al.* SciPy 1.0: Fundamental Algorithms for Scientific Computing in Python. *Nat. Methods* **17**, 261–272, DOI: 10.1038/s41592-019-0686-2 (2020).
